# Comparing the Effect of TGF-β Receptor Inhibition on Human Perivascular Mesenchymal Stromal Cells Derived from Endometrium, Bone Marrow and Adipose Tissues

**DOI:** 10.3390/jpm10040261

**Published:** 2020-12-01

**Authors:** Shanti Gurung, Daniela Ulrich, Marian Sturm, Anna Rosamilia, Jerome A. Werkmeister, Caroline E. Gargett

**Affiliations:** 1 The Ritchie Centre, Hudson Institute of Medical Research, Clayton, VIC 3168, Australia; Daniela.ulrich@medunigraz.at (D.U.); jerome.werkmeister@hudson.org.au (J.A.W.); caroline.gargett@hudson.org.au (C.E.G.); 2 Obstetrics and Gynaecology, Monash University, Clayton, VIC 3168, Australia; AnnaRosamilia@urogyn.com.au; 3 Department of Obstetrics and Gynaecology, Medical University of Graz, Auenbruggerplatz, 8036 Graz, Austria; 4 Cell & Tissue Therapies WA, Royal Perth Hospital, Perth, WA 6000, Australia; marian.sturm@health.wa.gov.au; 5 Centre for Cell Therapy and Regenerative Medicine, University of Western Australia, Perth, WA 6009, Australia; 6 Monash Health, Clayton, VIC 3168, Australia

**Keywords:** perivascular mesenchymal stromal cells, SUSD2, apoptosis, senescence, clonogenicity, endometrium, placenta, menstrual fluid, bone marrow, adipose tissue

## Abstract

Rare perivascular mesenchymal stromal cells (MSCs) with therapeutic properties have been identified in many tissues. Their rarity necessitates extensive in vitro expansion, resulting in spontaneous differentiation, cellular senescence and apoptosis, producing therapeutic products with variable quality and decreased potency. We previously demonstrated that A83-01, a transforming growth factor beta (TGF-β) receptor inhibitor, maintained clonogenicity and promoted the potency of culture-expanded premenopausal endometrial MSCs using functional assays and whole-transcriptome sequencing. Here, we compared the effects of A83-01 on MSCs derived from postmenopausal endometrium, menstrual blood, placenta decidua-basalis, bone marrow and adipose tissue. Sushi-domain-containing-2 (SUSD2^+^) and CD34^+^CD31^−^CD45^−^ MSCs were isolated. Expanded MSCs were cultured with or without A83-01 for 7 days and assessed for MSC properties. SUSD2 identified perivascular cells in the placental decidua-basalis, and their maternal origin was validated. A83-01 promoted MSC proliferation from all sources except bone marrow and only increased SUSD2 expression and prevented apoptosis in MSCs from endometrial-derived tissues. A83-01 only improved the cloning efficiency of postmenopausal endometrial MSCs (eMSCs), and expanded adipose tissue MSCs (adMSCs) underwent significant senescence, which was mitigated by A83-01. MSCs derived from bone marrow (bmMSCs) were highly apoptotic, but A83-01 was without effect. A83-01 maintained the function and phenotype in MSCs cultured from endometrial, but not other, tissues. Our results also demonstrated that cellular SUSD2 expression directly correlates with the functional phenotype.

## 1. Introduction

Perivascular mesenchymal stromal cells (MSCs) are a rare population of cells that self-renew and maintain homeostasis of the tissues in which they reside [1,2]. They are multipotent [3] and have immunomodulatory properties mediated by a paracrine mechanism through their secretory profile [4]. MSCs express major histocompatibility complex (MHC) I surface molecules but lack the expression of MHC II and costimulatory molecules CD80, CD86 or CD40 [5], culminating in their development and use for autologous (auto-) and allogeneic (allo-) treatments of immune-related disorders, a function that is distinct from their stem cell properties [6]. However, there is recent concern about the possibility of an acute antidonor immune response against infused MSCs following repeated allo-MSC administration [7,8,9,10]. Although MSCs are considered immunoprivileged, antidonor antibodies with a specificity to donor MHC II have been detected following allo-MSC administration, limiting the efficacy of the repeated use of allo-MSC [7,9,11,12]. Likewise, there are concerns regarding allo-MSCs differentiating in vivo and becoming more immunogenic, thereby activating the host immune system [9]. However, there is still no consensus on the advantages of using auto-MSCs over allo-MSCs from clinical trial outcomes to date. Nonetheless, the exciting discovery of MSCs in most tissues of the body has reinvigorated the discussion on using MSCs as an autologous cell source for therapies [13].

Human MSCs have been identified in many tissues [14], including bone marrow [15], adipose tissue [16], umbilical Wharton’s jelly [17], dermis [18], dental pulp of shed teeth [19], umbilical cord [17], placenta [20], menstrual blood [21] and pre- and postmenopausal endometrium [22,23,24]. Importantly, MSCs from the endometrium, identified as perivascular cells in the functional layer that is shed during menstruation, are also found in menstrual blood, as well as the decidua basalis of the maternal placenta. These tissues are easily harvested with minimal discomfort to patients and without anaesthesia, which is required for bone marrow and adipose tissue. Due to the ease of tissue procurement, MSCs from the endometrium, placenta and menstrual blood are actively being developed for clinical use and have been assessed for their reparative properties in preclinical animal models for a range of clinical conditions [25,26,27,28,29]. 

The International Society for Cellular Therapy (ISCT) defines MSC as plastic adherent-multipotent cells expressing CD105, CD73 and CD90 and lacking CD45, CD34, CD14, CD19 and HLA-DR surface molecules [30]. MSC also express distinct perivascular surface markers, such as Stro-1, CD271, CD146 and sushi-domain-containing-2 (SUSD2), that distinguish them functionally from the general stromal population in various tissues [13,29,31]. SUSD2 is a recently identified surface antigen that recognises highly clonogenic bone marrow MSCs [32,33] and clonogenic pre- and postmenopausal endometrial MSCs (eMSCs) [24,34]. In addition, although ISCT guidelines indicate CD34 as a negative marker for MSCs, recent studies have reported CD34^+^ multipotent perivascular progenitor populations in human bone marrow [35,36], adipose tissue [37,38] and skeletal muscle (satellite cells) [37,39,40]. In accordance with these findings, ISCT and the International Federation for Adipose Therapeutics and Science (IFATS) have recently published a joint statement defining CD34 as a marker for adipose tissue MSCs [37]. However, despite immunoselecting a pure population of MSC using these surface markers, extensive culturing of rare MSCs for clinical use is detrimental to the quality of cells due to spontaneous differentiation and senescence in cultures [41,42,43]. Furthermore, the quantity and quality of MSC decreases with increasing age [42,44,45]. 

The maintenance and expansion of MSCs during prolonged cultures require targeting pathways that regulate proliferation and spontaneous differentiation, such as transforming growth factor beta (TGF-β-) and platelet-derived growth factor (PDGF-) receptor pathways [46,47,48]. Previously, we showed that the small molecule A83-01, an Alk4/5/7 (TGF-β receptor) inhibitor, improved the potency of culture-expanded premenopausal eMSCs by promoting proliferation, increasing cloning efficiency and preventing replicative senescence and apoptosis, using functional assays and whole-transcriptome sequencing [46,49,50,51]. In this study, our aim was to determine if A83-01 has a similar effect in promoting proliferation and blocking apoptosis and senescence of MSCs from endometrial-derived tissues (postmenopausal (pmMSCs), menstrual blood (mbMSCs) and placental decidua basalis (pMSCs)) and compare this with MSCs derived from bone marrow (bmMSCs) and adipose tissue (adMSCs).

## 2. Materials and Methods

### 2.1. Ethics Approval and Consent to Participate

This study was conducted in accordance with the Declaration of Helsinki. All human tissues were collected following ethical approval from Monash Health and Monash University Human Research Ethics Committees (HREC). Postmenopausal endometrium was obtained as a part of a single arm phase IV clinical trial registered with the Therapeutic Goods Administration (CTNRN12610000563066). Menstrual blood and placentas were collected with approval from HREC 09270B, approved 29 October 2009, and 10103B, approved 1 July 2010, respectively. Adipose tissues were obtained with approval from HREC CF15/379-2015000185, approved 24 February 2015. Bone marrow stromal cells were provided by Cell & Tissue Therapies WA, Royal Perth Hospital (HREC approval EC 2012/015). All patients gave written informed consent.

### 2.2. Human Tissue Samples

Postmenopausal endometrium (*n* = 6) was obtained from women who took oestrogen replacement therapy (Progynova 2 mg/daily for 6–8 weeks) to regenerate the atrophic endometrium in order to obtain higher cell yields [24]. Menstrual blood samples containing fragments of shedding endometrium where eMSCs reside were collected from healthy women (*n* = 6) who were <40 years and not taking any hormones. Placentas were collected from women undergoing elective caesarean section (*n* = 6) at term pregnancy. Adipose tissues were obtained from healthy patients (*n* = 6) who were undergoing abdominal liposuction for cosmetic purposes. Bone marrow stromal cells from healthy donors (*n* = 6) were provided by Cell & Tissue Therapies WA, Royal Perth Hospital. Six samples were collected for each tissue type for this comparative study.

### 2.3. Isolation of MSCs 

#### 2.3.1. Postmenopausal Endometrial, Menstrual Blood, Placental Decidua-Basalis and Bone Marrow MSCs Using the SUSD2 Surface Marker

The pmMSCs were isolated using our previously published protocol [24]. Menstrual blood was collected in a menstrual cup on the second day of menstruation, filtered through a 40-µm sieve (BD Biosciences, San Diego, CA, USA) and the cell pellet was used to isolate mbMSCs according to our previously published protocol [34,46]. Approximately 5-mm-deep decidual tissue was collected from the central cotyledon of freshly collected placenta. Then, pMSCs were isolated using a similar protocol to pmMSCs. Bone marrow stromal cells were selected as plastic adherent cells and were received frozen at passage 2 or 3. Following thawing, SUSD2^+^bmMSCs cells were purified by magnetic bead sorting using previously published protocols [34,46]. 

#### 2.3.2. Adipose Tissue MSCs Using CD34^+^CD31^−^CD45^−^ Surface Markers

Two hundred millilitres of adipose tissue were washed with phosphate-buffered saline (PBS) and digested with 200-mL collagenase II (Worthington 330 U/mg dry weight, 1 mg/mL in PBS) in a shaking water bath for 1 h at 37 °C. The digested tissue was centrifuged at 800× *g* for 10 min and the stromal vascular fraction (SVF) pelleted. Leucocytes (CD45^+^) and endothelial (CD31^+^) cells were selected using CD45 Dynabeads (4 beads/cell, Invitrogen, Waltham, MA, USA) and CD31 Microbeads (Miltenyi Biotec, Bergisch Gladbach, North Rhine-Westphalia, Germany), respectively, according to the manufacturer’s protocols, and discarded. Finally, the negatively selected CD45^−^CD31^−^ cell population was incubated with CD34-PE antibody (BD Pharmingen, San Diego, CA, USA), followed by anti-PE beads (Miltenyi Biotec, Bergisch Gladbach, North Rhine-Westphalia, Germany), and CD34^+^ CD31^−^CD45^−^ adMSCs were collected by eluting through a magnetic column.

### 2.4. Propagation of MSCs 

Primary MSCs were cultured in Dulbecco’s modified Eagle’s medium (DMEM)-F12/10% foetal calf serum (FCS)/1% antibiotics in a tri-gas humidified incubator in 5% O_2_, 5% CO_2_ and 90% N_2_, with the media changed every 2 to 3 days. Once ~70% confluent, media was changed every 24 h to 5% and 1% FCS medium and, then, to serum-free medium with growth factors—10 ng/mL each of basic fibroblast growth factor (FGF2) and epidermal growth factor (EGF) (serum-free medium or SFM) that showed enhanced eMSC proliferation previously [52]. Following the second passage, cells were seeded at 5000 cells/cm^2^ onto fibronectin-coated plates with SFM changed every 2 to 3 days. bmMSCs could not be cultured in a DMEM-F12-based SFM; therefore, they were cultured in α-MEM with 10% FCS and 10-ng/mL FGF2 on uncoated plates. At P6, except for adMSCs (P4), MSCs for each tissue type were divided into two groups, which were treated with either 1-µM A83-01 (Tocris, Minneapolis, MN, USA) or 0.01% dimethyl sulfoxide (DMSO) vehicle (control) for 7 days, with the media changed every 48 h. Cells were then trypsinised with TrypLE^TM^ for phenotyping and functional assays.

### 2.5. Immunophenotyping MSCs

Harvested A83-01-treated and control cells were resuspended at 10^5^ cells/tube in 2%FCS/PBS. The cells were washed and incubated with PE-, APC- or FITC-conjugated primary antibodies and matched isotype controls for 30 min in the dark on ice. Primary antibodies used were CD146, CD73 and CD105; PE-conjugated antibodies were CD140b, CD34, CD31 and CD271, while APC-conjugated antibodies were SUSD2, CD45, CD44 and CD90, as detailed in Table 1. Isotype control antibodies at the same concentration as the primary antibody were included for each run and were used to set the electronic negative control gate to <1% positive cells on the flow cytometer. Samples were analysed using a Flow Cytometer (Canto-II, Beckman Coulter). The data were analysed using FlowJo 7.6.3.

### 2.6. Colony-Forming Assay

Following treatment with and without A83-01, cells were seeded at 50–100 cells/cm^2^ in a fibronectin-coated culture plate in SFM for three weeks with weekly media changes. The clones on the plates were fixed in 4% Paraformaldehyde (PFA) for 10 min, stained with haematoxylin (Amber Scientific) for 5 min after washing with PBS and transferred to Scott’s tap water to develop blue colour. Colonies with more than 50 cells were considered a colony. Cloning efficiency was calculated by counting the number of colonies and dividing by the total number of cells seeded and calculating the percentage [22].

### 2.7. Cell Cycle and Apoptosis Analyses by Flow Cytometry

To assess the cell cycle, P6 MSCs treated with and without A83-01 were trypsinised, pelleted and fixed/permeabilised in ice-cold 70% ethanol for 30 min at 4 °C. The cells were centrifuged at 2000 rpm for 5 min at 4 °C and washed with PBS. The pelleted cells were incubated with 50-µL RNase (100 µg/mL, # R4875, Sigma) at room temperature (RT) for 15 min to remove any RNA contamination. Propidium iodide (PI) (200 µL, 50 µg/mL, Sigma) was added and samples analysed immediately by flow cytometry using BD FACS Canto^TM^ II using the linear mode for PI. The data were analysed using the Dean-Jett-Fox model in FlowJo 7.6.3 [46,53].

To assess apoptosis following treatment, P6 MSCs were trypsinised and stained with an Annexin V-APC/PI kit following the manufacturer’s protocols (eBioscience, Waltham, MA, USA). The trypsinised cells were pelleted and washed in 2% foetal bovine serum (FBS)/PBS, followed by washing with binding buffer and centrifuging at 1100 rpm for 5 min at 4 °C. The pellet was incubated with 5-µL Annexin-V-APC in 100-µL binding buffer for 10 min at RT protected from light, followed by washing with binding buffer and resuspending with 5 µL of PI in 200-µL binding buffer. Events were acquired immediately by flow cytometry using BD FACS Canto^TM^ II and analysed with FlowJo 7.6.3 [46].

### 2.8. Polymerase Chain Reaction (PCR) for SRY Gene

P4 pMSCs were digested with lysis buffer (100-mM Tris, pH 7.4, 5-mM EDTA, 0.5% SDS and 200-mm NaCl) and proteinase K (100 µg/mL, Sigma) at 50 °C for one hour in a shaker. The suspension was centrifuged at 140,000 rpm for 10 min at RT. Genomic DNA (gDNA) was precipitated from the supernatant with an equal volume of isopropanol. This was followed by washing with 70% ethanol and drying any residue before resuspending in RNase-free water. PCR was carried out in a 20-µL volume consisting of 10-µL Mytaq (Bioline), 1 µL each of forward (5′-tcagcaagcagctgggatac-3′) and reverse (5′-aactgcaattcttcggcagc-3′) primers (10-µM SRY; Bioneer), gDNA and milli-Q water. The reaction consisted of initial denaturation at 95 °C for 2 min, followed by 30 cycles of denaturation for 30 s, annealing at 55 °C and extension at 72 °C for one min each. The PCR products were separated by 1.5% agarose gel electrophoresis. Concurrently, human eMSCs were used as a female negative control, while peripheral blood mononuclear cells from male donors were used as a positive control. PCR products were visualised by the ChemiDoc XRS+ system (Bio-Rad).

### 2.9. Immunofluorescence Microscopy

Human term placental tissue from the central cotyledon was fixed in 4% PFA for 24 h, washed with PBS and equilibrated with 30% sucrose at 4 °C for 24 h. The tissue was then embedded in the Optimal Cutting Temperature compound. Eight-micrometre tissue sections were blocked by protein block (Dako, X0909) for 1 h, followed by incubation with SUSD2-PE-conjugated antibody (5 µg/mL, clone W5C5; Biolegend) for 1 h at RT in dark. The isotype control IgG1 antibody was used at the same concentration as a negative control. Nuclei were stained for 5-min incubation with Hoechst 33258 (0.5 µg/mL; Molecular Probes). Images were visualised and photographed using a Nikon fluorescent microscope and analysed using ImageJ software (ImageJ/NIH).

### 2.10. Detection of Cell Senescence by β-Galactosidase Activity

Adipose tissue MSCs were cultured in SFM with and without A83-01 on fibronectin-coated coverslips, as described above. They were fixed with 4% PFA for 10 min and washed with PBS, followed by freshly prepared X-Gal (1 mg/mL in DMSO) staining reagent (5-mM K_3_(Fe[CN]_6_), 5-mM K_4_(Fe[CN]_6_), 2-mM MgCl_2_ and 150-mM NaCl) in sodium citrate buffer at pH 6 for 24 h at 37 °C in a nonhumidified incubator. The cells were washed twice with PBS and counterstained with nuclear fast red (#N3020, Sigma-Aldrich, 0.1% *w*/*v*) for 10 min. Images were taken with a DP25 digital camera (Olympus). 

### 2.11. Statistics

The data were not normally distributed (Shapiro-Wilk test). Nonparametric Wilcoxon matched-pairs test was used to test for statistical significance between untreated control and A83-01-treated groups. Data are presented as medians with interquartile ranges. Differences were considered statistically significant at *p* < 0.05. 

## 3. Results

### 3.1. Localisation and Selection of pMSCs in Decidua Basalis

**Figure 1:**
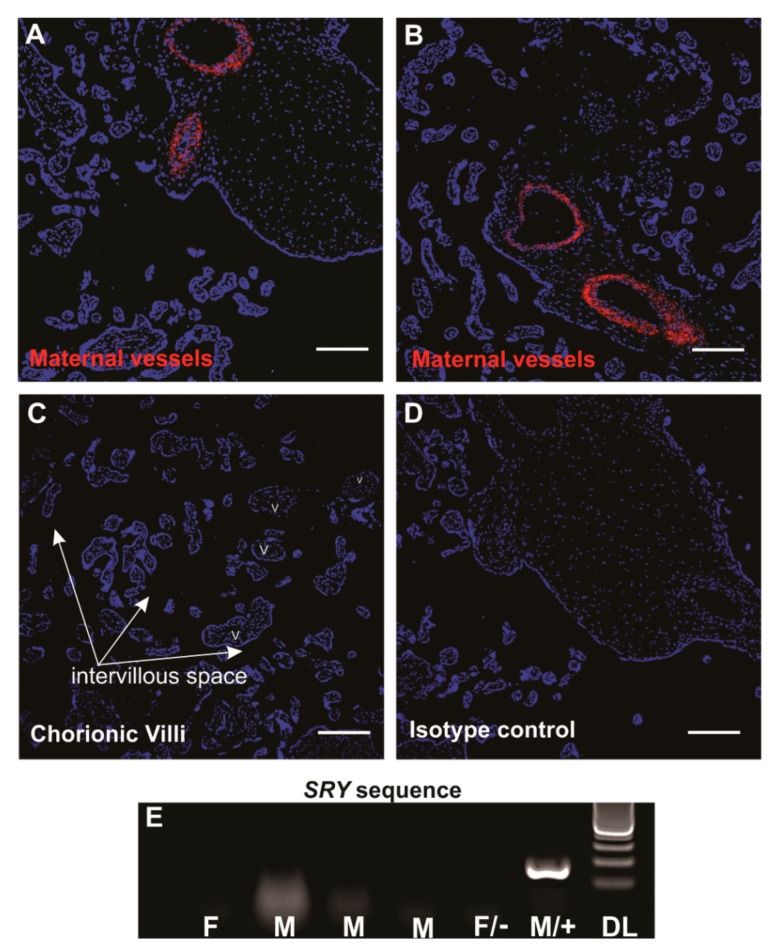
Sushi-domain-containing-2 (SUSD2^+^) mesenchymal stromal cells (MSCs) in human term placentae. Middle cotyledon from human term placental sections containing maternal vessels immunostained with SUSD2 (red) showing a perivascular location (**A**,**B**) with no immunostaining in the foetal chorionic villi. V, villi; intervillous space, arrows (**C**). Panel (**D**), isotype control. Scale bars = 200 µm. (**E**) PCR gel image showing no SRY genomic DNA (gDNA) bands in 4 placental decidua basalis MSC (pMSC) lines in the first four columns, indicating the absence of male foetal cells in the pMSC cultures. F, donor delivered female baby; M, donor delivered male baby; F/−, female negative control cells and M/+, male positive control cells for SRY primers. DL, 100-bp DNA ladder.

SUSD2^+^ eMSCs reside in a perivascular location in both functional (shed at menstruation) and basal layers of the endometrium [23,29,34]. We first looked for perivascular SUSD2^+^ cells in the central cotyledon of decidua basalis, because it is derived from the basal layer of the endometrium. Indeed, SUSD2^+^ cells were present around the maternal vessels in the decidua basalis (Figure 1A,B). No positive staining was detected in the chorionic villi and any foetal vessels towards the chorionic plate (Figure 1C) or in the isotype control (Figure 1D), indicating specificity for maternal pMSCs.

Of the six women who donated term placentae, three had given birth to healthy boys and one a healthy girl (gender of babies from two donors were unknown). To confirm noncontamination of foetal cells in our pMSC lines, we used a PCR assay to detect the male-specific SRY gene in gDNA. None of the pMSCs from male placentae showed a positive band for SRY (Figure 1E), confirming our method of isolated and propagated pure maternal origin pMSCs, as was established previously for unselected placental decidua basalis cells [54].

### 3.2. A83-01 Promotes MSC Proliferation

**Figure 2:**
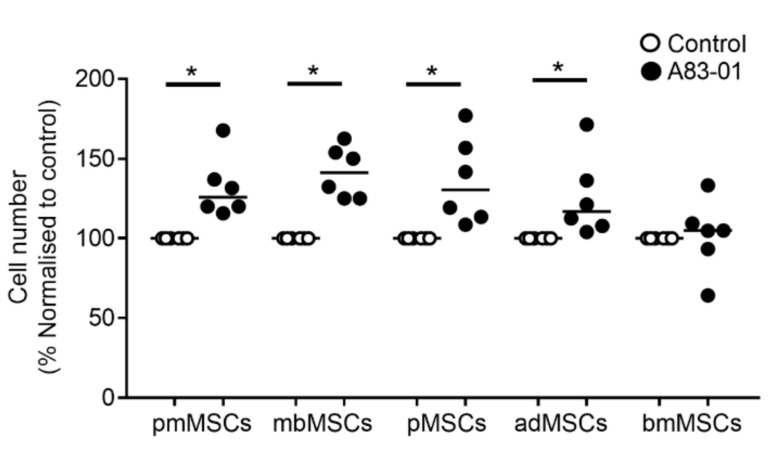
A83-01 promotes MSC proliferation. P6-cultured postmenopausal MSCs (pmMSCs), menstrual blood MSCs (mbMSCs), pMSCs and bone marrow MSCs (bmMSCs) and P4 adipose tissue MSCs (adMSCs) were treated with and without A83-01 in 5%O_2_/5%CO_2_/90%N_2_ for 7 days. The cell number obtained was normalised to the DMSO vehicle control (set to 100%) for *n* = 6 biological samples for each tissue type. Bars, medians. * *p* < 0.05.

In our previous study, we demonstrated that A83-01 promoted proliferation of P6 pre-menopausal eMSCs compared to vehicle controls [46]. To examine the effect of A83-01 on MSCs from other tissue sources, cultured MSCs were divided into two groups and treated with and without 1-μM A83-01 for 7 days and live cells were counted using trypan blue exclusion method. Similar to the effect on pre-menopausal eMSCs, A83-01 significantly promoted proliferation of the tested MSCs compared to controls (Figure 2, *p* < 0.05) however, this effect was not evident in bmMSCs (Figure 2).

### 3.3. Surface Phenotype of A83-01-Treated MSCs

**Figure 3:**
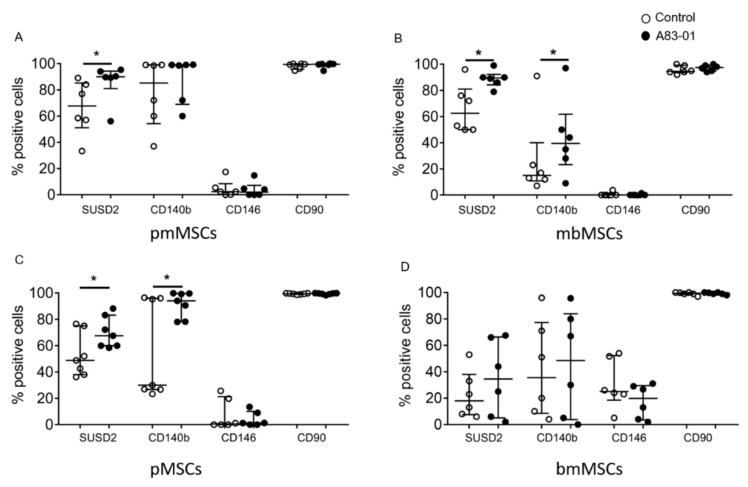
The surface phenotype of P6 MSCs cultured with and without A83-01 in 5%O_2_. The percentage of positive cells for MSC surface markers following the culture in 1-µM A83-01 (black circles) and 0.01% DMSO (white circles) for 7 days from (**A**) postmenopausal, (**B**) menstrual blood, (**C**) placenta decidua basalis and (**D**) bone marrow MSCs, assessed by single-colour flow cytometry. Data are plotted as medians with interquartile ranges of *n* = 6 biological samples for each tissue type. * *p* < 0.05.

**Figure 4:**
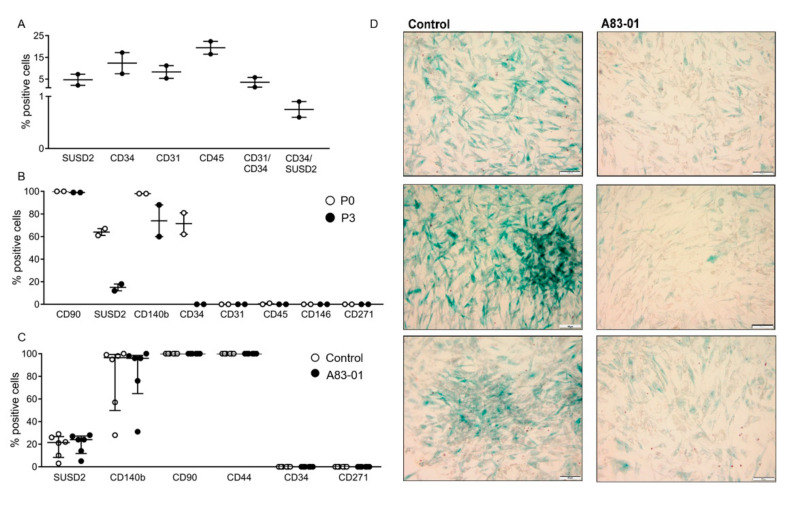
The surface phenotypic profile of freshly isolated and cultured adMSCs and senescence-associated β-Gal activity following the culture with and without A83-01 at P4 in 5%O2. (**A**) The percentage of positive cells for surface markers from freshly isolated adipose tissue stromal vascular fraction by flow cytometry. Graph shows individual results of *n* = 2 samples and the mean (bar). (**B**) Surface markers of P0 and P3 adMSCs by single-colour flow cytometry. Graphs show individual results of *n* = 2 and the mean (bar). (**C**) Phenotypic profile of surface markers on P4 adMSCs cultured with 1-µM A83-01 and control (0.01% DMSO) for 7 days assessed by single-colour flow cytometry. Data are medians with interquartile ranges of *n* = 6 biological replicates. (**D**) Representative images of three control and A83-01-treated adMSCs. adMSCs underwent replicative senescence with increasing lysosomal β-galactosidase activity demonstrated by blue staining, which was markedly decreased with the A83-01 treatment. Scale bar = 100 µm.

We next examined the phenotype of MSCs treated with and without A83-01. A single-colour flow cytometric analysis revealed all MSCs were >95% positive for CD90, a representative ISCT marker (Figure 3 and Figure 4B). As demonstrated previously for premenopausal eMSCs [46], the percentage of CD90^+^ MSCs did not change in MSCs from the postmenopausal endometrium, menstrual blood, placenta, bone marrow or adipose tissue following A83-01 treatment, confirming its ubiquitous presence in stromal cells, fibroblasts and MSCs [55]. Following A83-01 treatment, the percentage of SUSD2^+^ cells increased in MSCs from the postmenopausal endometrium, menstrual blood and placenta; however, an effect of A83-01 on the overall bmMSCs was highly variable and not evident (Figure 3D), although the percentage of SUSD2^+^ cells appeared either relatively low or high in different patient samples (Figure S1). As such, A83-01 increased the percentage of SUSD2^+^ cells from 29% to 60% in three bmMSCs, while there was little change in the other three (15% to 11%) (Figure S1A), with no change in cell numbers between the A83-01-treated and control cells in the high and low groups (Figure S1B). Similar to premenopausal eMSCs in our earlier studies [46], the percentage of CD140b^+^ cells also increased in menstrual blood and placental decidua basalis MSCs following A83-01 treatment, while there were no differences in the pmMSCs and bmMSCs, with the latter showing highly variable results. The most significant change observed was in the percentage of CD146^+^cells for all MSC types. It was very low in cultured MSCs, except for bmMSC cultures, which had a higher percentage of CD146^+^cells (30%), but A83-01 was without effect (Figure 3D).

A different isolation process was performed for adipose tissue based on the surface profile of adipose tissue-derived MSCs reported in the literature [37,38]. The adipose tissue stromal vascular fraction (SVF) consisted of 4.7% SUSD2^+^, 12.3% CD34^+^, 8.3% CD31^+^ and 19.5% CD45^+^ cells, with 3.5% of cells both CD31^+^ and CD34^+^, while 0.75% were both CD34^+^ and SUSD2^+^ (Figure 4A). The overlap of CD34^+^ cells in adipose tissue SVF with endothelial cells highlights the need for their negative selection to obtain a pure population of adMSCs. Surface marker profiles of adMSCs were also assessed following the culture of CD34^+^CD31^−^CD45^−^ adMSCs at P0 and P3. The percentage of CD90^+^adMSCs was high (Figure 4B) (100% at P0 and 98% at P3), similar to other MSCs, and did not change even at P4 (Figure 4C). Cultured adMSCs were negative for CD31, CD45, CD146 and CD271 surface markers (Figure 4B). At P0, 64% and 98% of adMSCs were SUSD2^+^ and CD140b^+^, respectively, which decreased to 15% and 74% by P3 (Figure 4B). Similarly, 71.5% of P0-cultured adMSCs were CD34^+^, but by P3, none were. At P4, 19% of adMSCs were SUSD2^+^, 80% CD140b^+^, 100% CD90^+^, 100% CD44^+^, 0% CD34^+^ and 0% CD271^+^, and there was no change in the percent of positive cells for all these surface markers following treatment with A83-01 (Figure 4C).

### 3.4. Analysis of MSC Senescence in A83-01-Treated and Untreated MSCs

Given the inability to propagate adMSCs beyond P4, senescence-associated lysosomal β-galactosidase (SAβ-Gal) was assessed, rather than undertaking apoptosis and cell cycle assays, by incubating the cells with X-Gal reagent at pH6, which we have previously shown for premenopausal eMSC [46]. As shown in Figure 4D, P4 control adMSCs displayed more blue staining, indicative of senescent cells, compared with A83-01-treated cells, which showed fewer blue senescent cells.

### 3.5. Effects of A83-01 on MSC Colony-Forming Unit Activity

**Figure 5:**
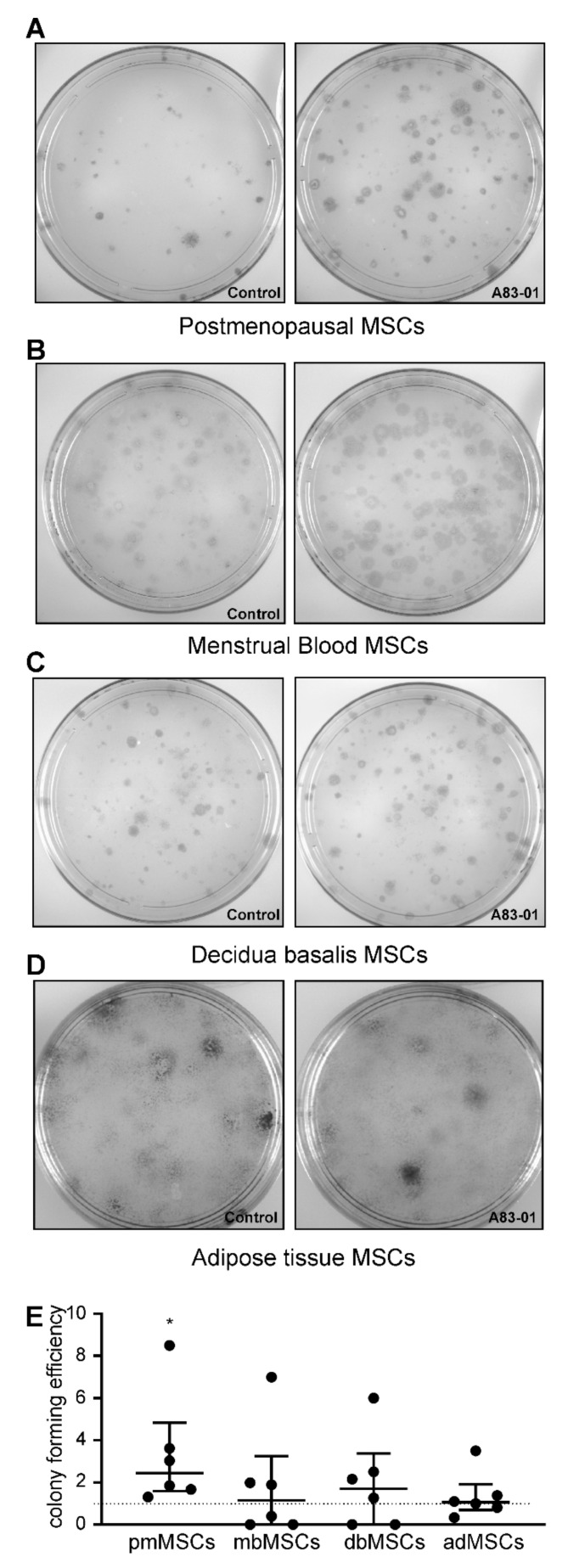
Colony-forming efficiency of MSCs treated with (treatment group, right panels) and without (control group, left panels) A83-01 in serum-free medium (SFM). (**A**–**D**) Representative culture plates of (**A**) postmenopausal, (**B**) menstrual blood, (**C**) placental and (**D**) adipose tissue MSCs seeded at a clonal density of 50–100 cells/cm^2^. (**E**) Graph showing colony-forming efficiency of P6 MSCs (pmMSCs, mbMSCs, pMSCs and P4 adMSCs pretreated with and without 1-µM A83-01 for 7 days, followed by low-density clonal culture in SFM for 3 weeks. The colony-forming efficiency was normalised to the corresponding control of each tissue. Data are medians with interquartile ranges of *n* = 6 different patient samples. * *p* < 0.05.

A colony-forming assay was used to assess the enrichment of MSCs in the A83-01-treated and vehicle control groups. There was a significant 3.3-fold increase in the colony-forming efficiency in pmMSCs treated with A83-01 (*p* < 0.05) (Figure 5A,E). However, there were no differences in colony-forming ability in the menstrual blood, placenta and adipose tissue MSCs treated with and without the small molecule A83-01 (Figure 5B–E). bmMSC were unable to form colony-forming units (CFU) at passage 6.

### 3.6. Effects of A83-01 on the MSC Cell Cycle and Apoptosis

**Figure 6:**
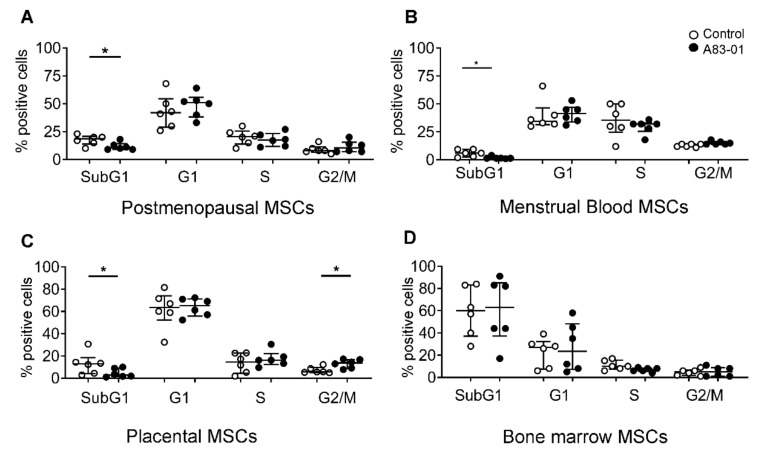
Cell cycle analysis of A83-01-treated and untreated pmMSCs, mbMSCs, pMSCs and bmMSCs by flow cytometry. The graphs show the percentages of (**A**) pmMSCs, (**B**) mbMSCs, (**C**) pMSCs and (**D**) bmMSCs in the SubG1, G1, S and G2/M phases of the cell cycle, following 7 days of culture in A83-01-containing SFM. Data are medians with interquartile ranges of *n* = 6 different patient samples. * *p* < 0.05.

To investigate the cell cycle stage profiles of MSCs treated with and without A83-01, PI-stained cells were assessed by flow cytometry. The treatment with A83-01 decreased the percentage of apoptotic cells (SubG1 phase) in MSCs from the postmenopausal endometrium, menstrual blood and placenta (Figure 6A–C). There was also a significant increase in the G2/M phase (mitotic cells) in the pMSCs treated with A83-01. Unlike all other MSCs tested, there were no differences in any cell cycle phase in bmMSCs, although 63% of cells were in the SubG1 phase in both A83-01-treated and the untreated control, indicating that a substantial proportion of cultured bmMSCs were apoptotic (Figure 6D).

**Figure 7:**
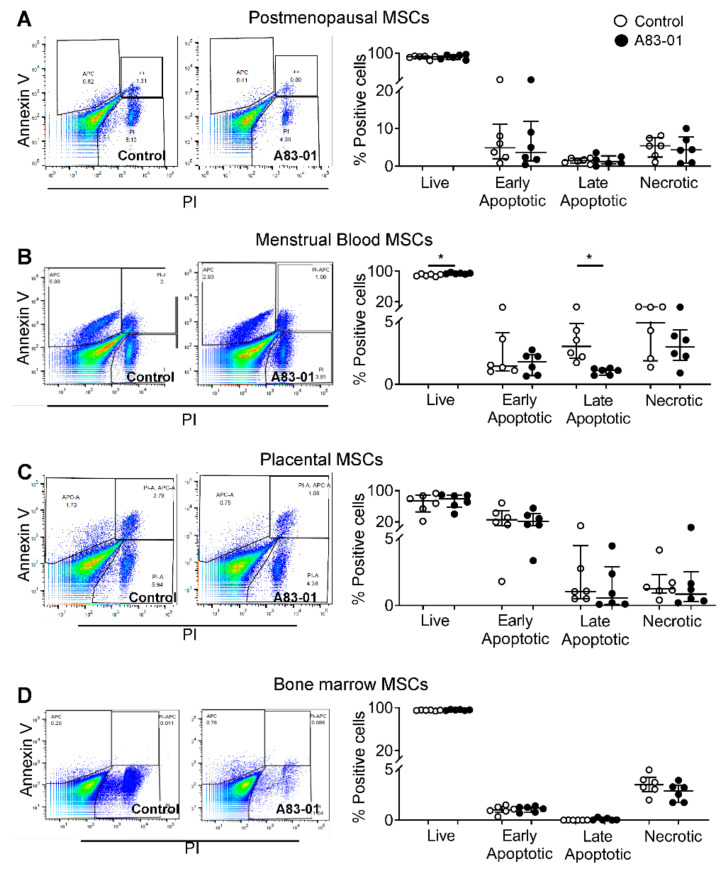
Analysis of apoptotic cells in A83-01-treated and untreated MSCs using Annexin V-PI. The graphs show the percentage of live (Annexin-V^−^/PI^−^), early (Annexin-V^+^/PI^−^) and late-apoptotic (Annexin-V^+^/PI^+^) and necrotic cells (Annexin-V^−^/PI^+^) in A83-01-treated and untreated MSCs from (**A**) pmMSCs, (**B**) mbMSCs, (**C**) pMSCs and (**D**) bmMSCs (while circles, control and black circles, A83-01). Left panels show representative flow cytometry dot plots for the control and A83-01 cells for each cell type. Data are medians with interquartile ranges of *n* = 6 different patient samples. * *p* < 0.05.

A flow cytometry analysis was also used to assess cell apoptosis by double-staining with Annexin-V and PI. Live (Annexin-V^−^/PI^−^), early apoptotic (Annexin-V^+^/PI^−^), late-apoptotic (Annexin-V^+^/PI^+^) and necrotic cells (Annexin-V^−^/PI^+^) in the cultures were quantified. Live cells comprised 88%, 90%, 67% and 95% in pmMSCs, mbMSCs, pMSCs and bmMSCs, respectively (Figure 7). There was a significant increase in live cells in mbMSC cultures (90% ± 1.3% in the control vs. 94% ± 0.7% in A83-01-treated, *p* < 0.05) and a decrease in the late-apoptotic cells (3.5% ± 0.7% in the control vs. 1.0% ± 0.1% in A83-01-treated, *p* < 0.05) (Figure 7B). However, there were no significant differences in either the live or apoptotic cells following A83-01 treatment for other MSCs (Figure 7A,C,D). In contrast to the large SubG1 population identified using the cell cycle analysis in bmMSCs (Figure 6D), 95% bmMSCs were identified as live cells by the Annexin V-PI assay.

## 4. Discussion

This study demonstrated that A83-01 significantly promoted MSC proliferation, increased the percentage of SUSD2^+^ MSCs and decreased apoptotic cells, especially in cultures derived from endometrium-derived tissues—postmenopausal endometrium, placental decidua basalis and menstrual blood. In these tissues, the various SUSD2^+^ MSC are the same as, or originated from, eMSCs. Cultured purified CD34^+^CD45^−^CD31^−^ adMSCs had limited ability to proliferate and showed exaggerated replicative senescence mitigated by A83-01, while bmMSCs underwent significant cellular apoptosis upon extensive culture. In this study, we also identified SUSD2^+^ MSCs in the placental decidua basalis and validated their maternal origin by demonstrating the absence of foetal cell contamination in our pMSC cultures. 

TGF-β (transforming growth factor beta) regulates a wide range of biological functions, including cell proliferation, differentiation and apoptosis. TGF-β is a common cytokine secreted by MSCs from bone marrow, adipose tissue and human endometrium [50,56]. It is a potent inhibitor of epithelial cell, keratinocyte, Lgr5^+^ liver and intestinal epithelial stem cells and lymphocyte and chronic lymphocytic leukaemia B cell proliferation [57,58,59,60,61]. Lgr5^+^ intestinal epithelial cells cultured in A83-01-containing medium had a significantly increased lifespan, colony-forming efficiency and percentage of Lgr5^+^ epithelial stem cells [57,60]. Therefore, our approach was to not completely inhibit the TGF-βR signalling pathway but to maintain it such that proliferation is maintained or increased but spontaneous differentiation and apoptosis are significantly inhibited, allowing the expansion of functional MSC. Herein, we demonstrated that A83-01, a TGF-β receptor inhibitor, increased the proliferation of long-term cultured MSCs from the postmenopausal endometrium, menstrual blood, placental decidua basalis and adipose tissue in a similar manner to premenopausal eMSCs [46], indicating that the endogenous synthesis of TGF-β had a negative effect on MSC growth during culture expansion in serum-free medium (SFM). However, this was not the case for bmMSCs, suggesting different signalling pathways are involved in MSC derived from bone marrow. While the concentration of A83-01 was optimised for culture in serum-free medium to inhibit autocrine TGF-β secreted by the cells, however, for bmMSC, the concentration may not be sufficient to block autocrine, as well the TGF-β, present in the serum medium required for their culture. A limitation of our study was that we did not use a commercial serum-free medium for bmMSCs, which may reveal a similar effect of A83-01 as for the other MSC types. Another limiting factor that could confound our findings were the different isolation methods used, as we specifically used currently published methods that differed for several MSC types.

In the present study, we chose one of the ISCT surface markers, CD90, as a representative MSC marker. As demonstrated in five different MSC types, CD90 is a ubiquitous protein on the surface of stromal cells. CD90 does not change during MSC propagation, and its expression does not reflect differences in the morphology and functional potency [38,62]. Other ISCT markers similarly showed that the percentage of positive cells did not change during the MSC culture [37,38,52]. SUSD2 is a recently identified surface marker for bmMSCs and clonogenic MSCs from the endometrium and other tissues [33,34,46,49]. However, the regulation of SUSD2 expression was unknown until our previous study showed that SUSD2 mRNA and protein is regulated through the TGF-β/SMAD2 pathways in eMSCs [46]. Here, we showed that the culture expansion of MSCs from various sources leads to a decrease in the percentage of SUSD2-expressing cells, confirming spontaneous fibroblast differentiation upon culturing, a well-known phenomenon in the MSC field [50,51,63,64,65]. The percentage of SUSD2^+^ cells from endometrial and endometrium-derived tissues increased following A83-01 treatment but not in adipose tissue and bone marrow MSCs. Herein, we also chose to further investigate the effect of A83-01 on MSC’s colony-forming (CFU) ability, a key stem cell property [15]. However, despite a significant increase in the proliferative ability and SUSD2^+^ cells, an increase in CFU activity was only evident in pmMSCs pretreated with A83-01. The low live (~62%) and SUSD2^+^ cell (~10%) contents of our starting bmMSCs following thawing may compromise the beneficial effect of A83-01 on the bmMSCs. Additionally, A83-01 may have a greater effect if the bmMSC was cultured in a commercial SFM rather than the traditional serum-containing medium. 

Recently, MSCs isolated from adipose tissue using the CD34 surface marker were found more clonogenic than those using the SUSD2 antibody [37,38]. However, these adMSCs isolated using CD34 had significant contamination with CD31^+^ endothelial cells and CD45^+^ leucocytes [38]. In our study, we removed red blood cells by the Ficoll density gradient, as well as endothelial cells and leucocytes by magnetic bead selection using their respective antibodies before purifying the CD34^+^ adMSC population according to the recently published joint statement by the ISCT and IFATS [37]. Despite this selection process, and as has been shown in another study [38,66], CD34 expression on adMSCs significantly decreased as early as the first passage, and there were no CD34^+^ cells by the third passage. This indicates that, despite due care in selecting pure adMSCs, they undergo rapid spontaneous fibroblast differentiation and senescence during culture expansion. Furthermore, here, adMSCs were cultured in physiological 5% O_2_ in a DMEM-F12-based SFM, while others either enriched with the CD34 antibody alone and cultured them with 10% serum medium in 20% O_2_ or cultured the SVF in serum or xeno-free medium [38,67,68]. The chronic inflammatory milieu in vivo in obese donors can have a negative impact on adMSCs’ functional and therapeutic potentials [69]. These differences in enriching and culturing, together with the varied inflammatory tissue environment, could limit the effect of A83-01 on adMSCs, thereby contributing to inconsistent results between ours and other studies. Nonetheless, adMSCs underwent senescence quite early in the culture, indicating an impaired or loss of function. The resultant heterogeneity of the cultures resulting from varied culture expansion protocols highlights the need for consistency in culture expansion protocols of adMSCs for clinical translation and, indeed, for MSC from all tissue types [70]. Characterising the effects of our SFM containing A83-01 for endometrial and endometrium-derived MSC is an important step in developing them for clinical use. 

TGF-β is a pleiotropic cytokine that mediates stem cell differentiation, especially into smooth muscle cells [71,72]. TGF-β also promotes senescence in several cell types [72,73,74] and induces apoptosis via upregulating death-associated protein kinases and the TGF-βR-inducible transcription factor [75,76,77]. Using two approaches, we demonstrated that A83-01 prevented apoptosis, especially in MSCs from endometrial and endometrium-derived tissues, and the senescence of adMSCs. Interestingly, the cell cycle analysis of bmMSCs showed 63% of SubG1 apoptotic cells in contrast to 95% of live cells using the Annexin-V/PI analysis. The cell cycle analysis uses 70% ethanol to permeabilise the cell/nuclear membranes, enabling any fragmented DNA multimers (from dead/dying cells) to leak out, reducing the cellular DNA content and resulting in less DNA to bind with PI and an apparent increase in the apoptotic cells (SubG1) profile. In contrast, the Annexin-V/PI protocol relies on the integrity of the cell membrane and detects early apoptotic cells later than those with a loss of DNA integrity. Therefore, the permeabilization process allows for the true identification of apoptotic cells in otherwise healthy-looking cells [78]. This may account for the differences we observed in the Annexin-V/PI and cell cycle assays for bmMSCs, thus giving a more accurate assessment of the bmMSC status in the culture. This indicates the importance of applying multiple approaches to analyse cells to understand their functions in vivo. 

We and others have demonstrated that the cellular expression of SUSD2 has a role in preventing senescence and cell death [46,50,79], supporting its use as a surface marker to monitor MSC cultures and its potential as a potency assay for MSC production for cell therapies. The SUSD2 expression in cultured endometrial-derived MSCs (pre- and postmenopausal, menstrual blood and placental decidua basalis); adMSCs and bmMSCs highlights the value of this surface marker for monitoring MSC cultures. SUSD2 functions in preventing apoptosis and senescence [46,80], which may explain the increased senescence in adMSCs and cell death in bmMSCs with a decreasing SUSD2 expression over the culture period. Our findings also emphasise the importance of SUSD2 as the most reliable and sensitive marker of eMSCs. In future studies, the culture expansion of MSCs continuously with A83-01 from culture initiation may demonstrate improved effects in maintaining proliferation and preventing apoptosis and senescence for both endometrial and endometrium-derived bone marrow and adipose tissue MSCs. Promoting the proliferation of MSCs from any source, while controlling and preventing apoptosis and spontaneous differentiation into unwanted fibroblasts, will promote the generation of pure undifferentiated potent MSCs. In addition to A83-01, other small molecules targeting additional signalling pathways involved in spontaneous MSC differentiation into fibroblasts could be assessed in the future to mitigate the unwanted consequences of culture expansion and generate a homogeneous population of undifferentiated MSCs [70]. This study is also the first to demonstrate the presence of maternal SUSD2^+^ perivascular MSCs and the feasibility of their isolation from term placenta and menstrual blood. Furthermore, women like the concept of using MSCs from different tissues, especially from reproductive tissues, as autologous sources [81], and a culture expansion with A83-01 may provide an approach for generating enough undifferentiated MSCs for therapeutic applications. 

## 5. Conclusions

In conclusion, TGF-βR signalling is involved in cell fate in perivascular MSC from reproductive tissues, bone marrow and adipose tissue. We have shown that MSC undergo culture-induced spontaneous differentiation leading to loss of functions and that these events can be mitigated by targeting the TGF-βR using A83-01 as an additive in the culture medium. In addition to our previous findings with pre-menopausal eMSC, this study further supports to the concept that small molecules such as A83-01 can be used to propagate functional-MSC for cell-based therapies.

## Figures and Tables

**Table 1:** Antibodies used.

Primary Antibodies				
Antigen	Clone	Isotype	Conc. μg/mL	Supplier
CD73	AD2	Mouse IgG1	10	BD Pharmingen, San Diego, CA, USA
CD105	266	Mouse IgG1	10	BD Pharmingen, San Diego, CA, USA
CD146	CC9, [23]	Mouse IgG2a	1:1	A kind gift from Prof D Haylock CSIRO
CD140b	PR7212	Mouse IgG1	25	R&D systems, Minneapolis, MN, USA
CD34	581	Mouse IgG1	200	BD Pharmingen, San Diego, CA, USA
CD31	WM59	Mouse IgG2a	10	BD Pharmingen, San Diego, CA, USA
CD271	ME20.4-1.H4	Mouse IgG1	100	Miltenyi Biotec, Bergisch Gladbach, North Rhine-Westphalia, Germany
SUSD2	W5C5	Mouse IgG1	50	Biolegend, San Diego, CA, USA
CD45	HI30	Mouse IgG1	10	Invitrogen, Waltham, MA, USA
CD44	IM7	Rat IgG2b	10	eBioscience, Waltham, MA, USA
CD90	5E10	Mouse IgG1	25	BD Pharmingen, San Diego, CA, USA
PE rat anti-mouse IgG1		A85-1	2	BD Pharmingen, San Diego, CA, USA
FITC rat anti-mouse IgG2a		R19-15	5	BD Pharmingen, San Diego, CA, USA

## Supplementary Materials

The following are available online at https://www.mdpi.com/2075-4426/10/4/261/s1. Figure S1: Differences between bmMSC donors.
