# Supplementary material for: Comparing the Effect of TGF-β Receptor Inhibition on Human Perivascular Mesenchymal Stromal Cells Derived from Endometrium, Bone Marrow and Adipose Tissues
Source: J Pers Med. 2020 Dec 1;10(4):261. doi: 10.3390/jpm10040261 (PMC7712261; doi:10.3390/jpm10040261)
Supplement: Supplementary file 1 [file jpm-10-00261-s001.pdf]

Comparing the effect of TGF-β receptor inhibition on human mesenchymal stem/stromal cells derived from endometrium, bone marrow and adipose tissues

Shanti Gurung [1,2*], Daniela Ulrich [1,6], Marian Sturm [3,4], Anna Rosamilia [2,5], Jerome A. Werkmeister [1,2] and Caroline E. Gargett [1,2]

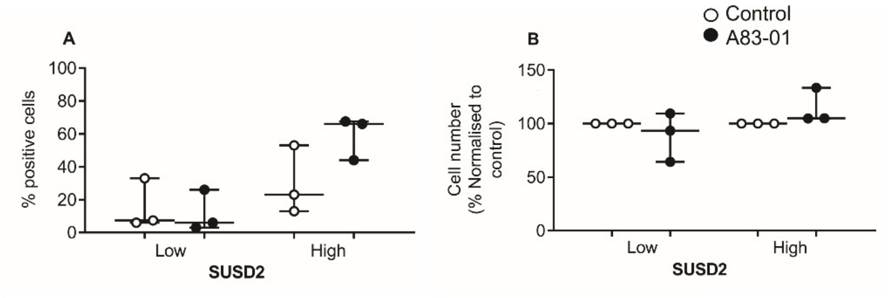

Figure S1. Differences between bmMSC donors. (A) Graph showing two groups of bmMSCs with and without effect of A83-01 treatment on % SUSD2⁺ cells. (**B)** Graph showing no difference in the number of cells following A83-01 treatment in the two groups of donor cells from **A**. Plots are median for n=3 biological samples per treatment group.
